# Evaluation of a Diabetes Self-Management Program: Claims Analysis on Comorbid Illnesses, Health Care Utilization, and Cost

**DOI:** 10.2196/jmir.9225

**Published:** 2018-06-22

**Authors:** Ralph M Turner, Qinli Ma, Kate Lorig, Jay Greenberg, Andrea R DeVries

**Affiliations:** ^1^ HealthCore, Inc Wilmington, DE United States; ^2^ Stanford Patient Education Research Center Palo Alto, CA United States; ^3^ National Council on Aging Services Arlington, VA United States

**Keywords:** diabetes mellitus, patient education, health care utilization, cost

## Abstract

**Background:**

An estimated 30.3 million Americans have diabetes mellitus. The US Department of Health and Human Services created national objectives via its Healthy People 2020 initiative to improve the quality of life for people who either have or are at risk for diabetes mellitus, and hence, lower the personal and national economic burden of this debilitating chronic disease. Diabetes self-management education interventions are a primary focus of this initiative.

**Objective:**

The aim of this study was to evaluate the impact of the Better Choices Better Health Diabetes (BCBH-D) self-management program on comorbid illness related to diabetes mellitus, health care utilization, and cost.

**Methods:**

A propensity score matched two-group, pre-post design was used for this study. Retrospective administrative medical and pharmacy claims data from the HealthCore Integrated Research Environment were used for outcome variables. The intervention cohort included diabetes mellitus patients who were recruited to a diabetes self-management program. Control cohort subjects were identified from the HealthCore Integrated Research Environment by at least two diabetes-associated claims (International Classification of Diseases-Ninth Revision, ICD-9 250.xx) within 2 years before the program launch date (October 1, 2011-September 30, 2013) but did not participate in BCBH-D. Controls were matched to cases in a 3:1 propensity score match. Outcome measures included pre- and postintervention all-cause and diabetes-related utilization and costs. Cost outcomes are reported as least squares means. Repeated measures analyses (generalized estimating equation approach) were conducted for utilization, comorbid conditions, and costs.

**Results:**

The program participants who were identified in HealthCore Integrated Research Environment claims (N=558) were matched to a control cohort of 1669 patients. Following the intervention, the self-management cohort experienced significant reductions for diabetes mellitus–associated comorbid conditions, with the postintervention disease burden being significantly lower (mean 1.6 [SD 1.6]) compared with the control cohort (mean 2.1 [SD 1.7]; *P*=.001). Postintervention all-cause utilization was decreased in the intervention cohort compared with controls with −40/1000 emergency department visits vs +70/1000; *P*=.004 and −5780 outpatient visits per 1000 vs −290/1000; *P*=.001. Unadjusted total all-cause medical cost was decreased by US $2207 in the intervention cohort compared with a US $338 decrease in the controls; *P*=.001. After adjustment for other variables through structural equation analysis, the direct effect of the BCBH-D was –US $815 (*P*=.049).

**Conclusions:**

Patients in the BCBH-D program experienced reduced all-cause health care utilization and costs. Direct cost savings were US $815. Although encouraging, given the complexity of the patient population, further study is needed to cross-validate the results.

## Introduction

### Background

Current national estimates indicate that 9.4% of Americans (30.3 million people) have diabetes mellitus (DM) [1-3]. Annual (2012) DM-related expenditures are estimated at US $245 billion, with US $176 billion in direct medical costs and US $69 billion in indirect costs attributable to disabilities, work absences, and premature death, among others [1-3]. Looking beyond the economic issues, having DM as well as common comorbid conditions such as hypertension and depression negatively impact quality of life (QoL), decrease functioning and ability to manage self-care, and raise mortality risk [4-11]. In response, the US Department of Health and Human Services created national objectives via its Healthy People 2020 initiative to help improve the QoL of people who either have or at risk for DM, and hence, lower the personal and national economic burden [12].

Diabetes Self-Management Education (DSME) interventions, which focus on guiding DM management and promoting healthy behaviors and lifestyle changes, are a primary part of this initiative. Many such interventions have been reported. Pal and colleagues completed a meta-analysis of 16 computer-based interventions (delivered through clinics, the internet, and mobile phones) and found small effects on glycemic control and no effects on depression or QoL [13]. Sherifali et al conducted an internet-based diabetes lifestyle management program and found improvements in body mass index, body fat percentage, and activity levels in participants [14]. A systematic review of nine internet-based diabetes lifestyle modifications demonstrated improvements in diet or activity level (2 studies) and improvements in glycemic control (2 studies) [15]. A meta-analysis of 13 studies using community health workers as interventionist found a modest reduction in glycated hemoglobin (HbA_1c_) compared with usual care [16]. A more recent meta-analysis for group-based intervention involving 47 studies concluded that group-based programs showed better outcomes when taught by one or more professionals with or without a peer when compared with peer-led education [17]. It should be noted that these studies illustrate different aspects of the current intervention—computer-based, internet-based, peer-led and theory-based, all of which are aspects of the intervention discussed in this paper.

There have also been several diabetes education cost-effectiveness studies. A recently completed meta-analysis of 8 studies found that 4 were based on reduction of clinical measures and 4 on quality-adjusted life years (QALYs) [18]. A program in South Africa that lowered blood pressure was reported as cost-effective based on QALYs gained [19]. Similar findings were found with a nurse-delivered telecoaching program in Belgium [20]. Evaluations of diabetes counseling and education programs have produced mixed economic results. Although most have shown a reduction in overall health care costs, not all programs have produced positive changes [21,22]. Telecoaching programs, including Web-based and phone-based programs, show particular promise in improving diabetes care and costs [23]. In a systematic review of interactive computer technology interventions to improve diabetes care, Jackson et al found an overall positive impact on diabetes care measures and health care utilization [24]. More recently, a quasi-experimental study by Nundy et al within an employer health plan found that mobile phone–based diabetes education was able to produce positive behavioral changes, improve glycemic control, and lower overall medical costs [25].

The intervention reported on in this study, Better Choices Better Health Diabetes (BCBH-D) program, originally developed at Stanford University, is an intensive DSME series of 2.5-hour sessions over 6 consecutive weeks that has demonstrated effectiveness [26]. The program was first evaluated as small groups in community settings near patients’ homes. More recently, online workshops have been developed and offered to persons with diabetes who preferred online communication because they lived in rural areas, wanted anonymity, were homebound, or had busy schedules [27]. The online program contains all the elements of the small group method, except participants log in and do the work from their personal computers. Participants complete exercises, read posted materials, and interact virtually with others in their group. Two related studies evaluating the small group and internet-based BCBH-D program, led by a consortium of the Stanford Patient Education Research Center, National Council on Aging (NCOA), and Anthem Health Plans, reported modest benefits among the program participants based on 6- and 12-month follow-up periods [28,29]. The 6-month follow-up study reported statistically significant improvements in 13 of 14 outcome measures, including HbA_1c_ and health behaviors (eg, communication with physicians, stretching or strengthening exercise, medication adherence, and frequency of eye, foot, cholesterol, and kidney exams) [28]. In the 12-month follow-up program, more than two-thirds (69.7%, 597/857) of the baseline study population experienced statistically significant improvements in 13 of the 15 prespecified outcome measures. The researchers noted that that the improvements at 6 months were maintained and augmented during the 12-month period [29]. However, to date, there has been no research that has evaluated the impact of the BCBH-D self-management program on health care utilization and costs.

### Objective

This study was designed as an administrative claims-based observational evaluation of the BCBH-D self-management program first offered in October 2013 to Anthem health plan enrollees in a real-world setting [28,29]. The objective of the present research was to evaluate (1) The program’s impact on comorbid illness related to DM, (2) Health care utilization, and (3) Health care costs within 12 months after program enrollment, compared with a propensity score matched control cohort of DM patients who received usual care but did not participate in the BCBH-D program. In addition, the study sought to better understand how baseline comorbid illness burden, age, gender, and prior health care costs influenced or mediated the 12-month changes in health care costs and if these differed by intervention and control groups.

## Methods

### Design

A propensity score matched two-group, pre-post observational design was used for this study. Retrospective administrative medical and pharmacy claims data from the HealthCore Integrated Research Environment (HIRE) were used for outcome variables. Administrative claims data comprehensively contains patient’s use of medical and pharmacy services, including hospitalization; emergency room; services occurring in an outpatient setting, such as office visits and laboratory test; and prescription fills. Researchers accessed a limited dataset in a Health Insurance Portability and Accountability Act compliant manner. Central Institutional Review Board (IRB) approval was obtained before the initiation of the study.

### Participants

The intervention cohort included DM patients who were recruited from October 2013 to October 2014 to participate in the program (reported on by Lorig et al), attended at least one session, and were identified in the HIRE claims database [29]. For inclusion in the BCBH-D, the participants were required to speak English and provide IRB-approved informed consent and were not permitted to have previously participated in a self-management program developed at Stanford. Methods and results of the active intervention have been previously reported [27-29]. For inclusion in this study, patients had to be found in the health care claims data with enrollment in the health plan for 1 year before and 1 year following the intervention. The index date for intervention cohort members was defined as the program enrollment date. Control cohort subjects were identified from the HIRE by at least two claims associated with diabetes (International Classification of Diseases-Ninth Revision, ICD-9 250.xx) within 2 years before the program launch date (October 1, 2011-September 30, 2013). Controls were 3:1 propensity score matched based on age, gender, health plan type, residence region, Metropolitan Statistical Areas (MSA or non-MSA), Deyo-Charlson Comorbidity Index (DCI) score (range: 0-25), comorbid illnesses, health care utilization, and total medical cost at 1-year baseline [30]. The index date was defined for controls as a randomly selected date during the same period as the BCBH-D program recruitment period (October 2013-October 2014). As the data for the control cohort was a limited dataset, a waiver was sought and obtained from the IRB, and informed consent was not required.

Patients in both cohorts were required to be members of an Anthem-affiliated health plan, aged 18 years or older, with type II diabetes, and to have continuous medical eligibility for 1 year before and after the index date. Exclusion criteria included major treatment for cancer (radiation, chemotherapy, or surgery) or pregnancy.

### Measures

Demographic measures included the preintervention values of gender, age, and geographical region of residence. DCI score was calculated to measure overall preintervention illness burden. Other clinical measures included a series of dichotomous variables documenting the absence or presence of 15 DM-associated comorbid illnesses, including hypertension, hyperlipidemia, obesity, chronic obstructive pulmonary disease, renal disease, depression, metabolic syndrome, ischemic heart disease, coronary heart disease, osteoporosis, osteoarthritis, lower back pain, peripheral vascular disease, musculoskeletal disorder, and sleep apnea, based on ≥2 ICD-9 codes for each (recorded in the outpatient; inpatient; or emergency department, ED setting) [30]. The 15 comorbid conditions were also measured during the follow-up year. In addition, an overall composite index that aggregated these 15 diseases at both pre- and postintervention periods was calculated for each patient to capture DM-associated comorbid illnesses burden.

Outcome measures included pre- and postintervention all-cause and diabetes-specific utilization and costs recorded in the HIRE claims database. Diabetes specific utilization was defined as hospitalizations and ED visits with primary diagnosis of diabetes and outpatient services (including but not limited to office visits, imaging, laboratory tests, and procedures) with any diabetes diagnosis on the claim. All-cause utilization was defined as any claims-based health care utilization inclusive of diabetes and any other diagnosis on the claim. All-cause and diabetes specific utilization measures for the 12-month pre- and postintervention periods encompassed hospitalizations, ED visits, and outpatient services, reported as visits per 1000 patients. All-cause and diabetes-specific costs were measured for overall medical services and for each service type, including hospitalizations, ED visits, and outpatient services. All-cause pharmacy costs were assessed for the 12-month pre- and postintervention periods.

### Statistical Analysis

The demographic and clinical characteristics at baseline were reported by percentage or mean for the Intervention and matched control cohorts. Chi-square tests and *t* tests were conducted to examine the differences between two cohorts. Repeated-measures analyses were conducted using the generalized estimating equation (GEE) approach, using a binomial distribution and a logit link for presence of comorbid conditions for objective 1, a negative binomial distribution and a logit link for utilization for objective 2, and a gamma distribution with log link for costs for objective 3. All costs are reported as least squares values. The GEE analysis allowed for two main independent comparisons. First, a statistical test assessed the overall cohort × time interaction that determined if the cohorts had different slopes (ie, different changes in outcome measures) between pre- and postintervention periods. Second, the test for a main effect among cohorts was divided into a comparison between the two cohorts at preintervention period and a comparison between the cohorts for differences at postintervention period. To better understand the cohort difference in the 12-month changes of health care costs controlling for the influence of baseline comorbid illness burden, age, gender, and prior health care costs groups, a maximum likelihood structural equation model (SEM) analysis was conducted.

**Figure 1:**
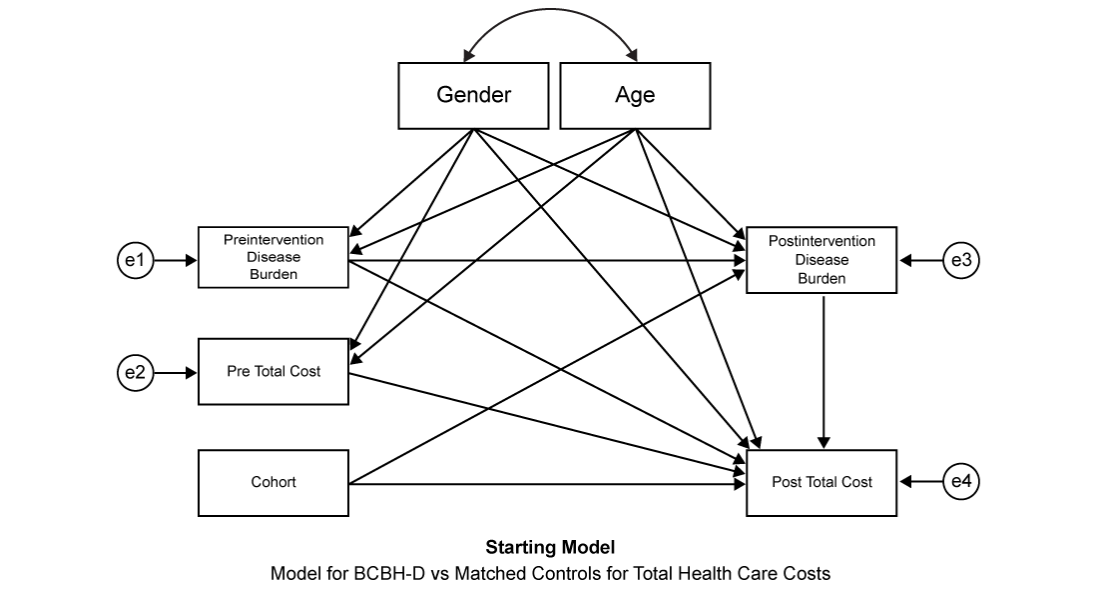
Starting model for structural equation model (SEM) analysis.

SEM was used because it permits a mediation analysis to identify and explain the process that accounts for the relationship between the treatment effect and total medical cost via the inclusion of a third variable, a mediator variable, which in this study is the composite index of DM-associated comorbid illnesses. This permits a test that changes in the composite index of DM-associated comorbid illnesses mediates a portion of the total postintervention medical costs.

Although SEM allows for the use of latent or unobserved variables, this study used only manifest measures. The starting model is shown in Figure 1. It was assumed that in addition to a cohort effect, patient gender, age, comorbid illness burden, and preintervention, total medical cost might account for postintervention total cost. As propensity score matching was used in this study, it was assumed that the cohort independent variable was uncorrelated with the sex, preintervention total costs, and preintervention comorbid illness burden. However, as treatment might impact on postintervention comorbid illness burden, this variable was assessed for its mediation effects.

Because the chi-square test of absolute model fit is sensitive to sample size and non-normality in the underlying distribution of the input variables, we used several descriptive fit statistics to assess the overall fit a model to the data, including the Comparative Fit Index (CFI), the Normed Fit Index (NFI), the Akaike Information Criterion (AIC), and the root mean square error (RMSE), indices to aid in judging the quality of model fit [31-33]. Statistical significance was defined as *P*<.05. Analyses were conducted using SAS Institute’s SAS Software, Version 9.3, except for the SEM, which was conducted in Stata, Version Release 14 by StataCorp [34,35].

## Results

### Identification of Clinical Study Patients in Health Care Claims Data

There were 1229 small-group and online participants in the Lorig et al (2016) study [29]. Within this population, 8 (0.65%) had no health insurance, 183 (14.89%) had Medicare, 2 (0.16%) were enrolled in Medicaid, 1 (0.08%) had Supplemental Security Income, 11 (0.90%) had Veterans benefits, 1014 (82.51%) had private insurance, and the insurance status of 10 study participants was unknown. We were able to identify 558 (55.03%, 558/1014) of privately insured) in the HIRE claims database who met the study criteria and also were covered for a full year before and after the participation date. The inability to identify the rest was probably because while participants were covered by an Anthem plan when they entered the study, they were not covered for a full year before and after the index date. In other cases, participants were enrolled in a Medicare supplemental plan where full health care utilization data were not available.

We compared the characteristics of the patients identified in claims (N=558) with the study participants we could not identify (N=671) to assess the potential for bias in our sample. The results are presented in Table 1. The majority of claims-identified patients (528/558, 94.6%) participated in the online workshop format and 28 (5.0%, 28/558) participated in the small-group format, whereas, 229 (18.6%, 229/1229) of all BCBH-D study participants chose the small-group program in the original study [29]. Claims-identified patients had a larger percentage of females, fell more heavily into the 41 to 64 years age categories, had a higher white than black racial composition, and self-reported a smaller arthritis comorbid illness burden than the patients that were not identified in claims.

**Table 1:** Characteristics of claims-identified study participants vs nonidentified study participants.

Characteristic		BCBH-D^a^ found in claims	BCBH-D not found in claims	*P* value	*R* ^2^
Total patients, n		558	671		
Female, n (%)		359 (64.5)	205 (30.5)	.001	.10
**Age categories (years), n (%)**				.001	.04
	21-30	3 (0.5)	35 (0.6)		
	31-40	25 (4.5)	95 (5.6)		
	41-50	133 (23.8)	336 (15.2)	<.05^b^	
	51-64	335 (60.4)	154 (53.8)	<.05^b^	
	≥65	61 (10.9)	624 (24.7)	<.05^b^	
**Race, n (%)**				.002	.02
	American Indian or Alaska Native	4 (0.7)	7 (1.0)		
	Asian (includes Indian)	22 (0.04)	22 (3.3)		
	Black or African-American	51 (9.1)	115 (17.1)	<.05^b^	
	Native Hawaiian or Pacific Islander	1 (0.2)	1 (0.1)		
	White (includes Hispanic or Latino)	466 (83.5)	504 (75.0)	<.05^b^	
	Two or more races	11 (2.0)	20 (3.0)		
	Declined	3 (0.5)	3 (0.4)		
Hispanic, n (%)		43 (7.7)	52 (7.7)	.99	.000
Married, n (%)		400 (71.7)	470 (69.9)	.42	.000
Years of education, mean (SD)		15.4 (2.7)	15.3 (2.8)	.52	.001
**Study status, n (%)**					
	Completed post measures	545 (97.7)	655 (97.5)	.29	.05
	Died	0 (0.0)	3 (0.4)		
	Lost to follow-up	12 (2.2)	14 (2.1)		
**Study treatment mode, n (%)**				.001	.09
	Online	528 (94.6)	473 (70.4)	<.05^b^	
	Small group	30 (5.4)	199 (29.6)	<.05^b^	
**Self-reported comorbidities, n (%)**					
	None	104 (18.6)	117 (17.4)	.66	.000
	Hypertension	340 (60.9)	418 (62.2)	.68	.000
	Asthma	51 (9.2)	54 (8.0)	.48	.004
	Arthritis	96 (17.2)	164 (24.4)	.002	.008
	Coronary heart disease	56 (10.1)	73 (10.9)	.65	.000
	Chronic obstructive pulmonary disease	19 (3.4)	18 (2.7)	.45	.000
	Cancer	27 (4.8)	32 (4.8)	.94	.000
	Renal disease	16 (2.9)	28 (4.2)	.22	.001
	Depression	103 (18.5)	127 (18.9)	.86	.000
	Mental health problems	68 (12.2)	77 (11.5)	.69	.000
**HbA_1c_^c^**		**N=432 (with valid test result)**	**N=523 (with valid test result)**		
	Pre, mean (SD)	8.29 (1.62)	8.12 (1.48)	.10	.00
	Post, mean (SD)	7.67 (1.32)	7.54 (1.41)	.28	.00
**Patient Health Questionnaire depression score**		**N=555**	**N=670**		
	Pre, mean (SD)	5.85 (4.75)	6.19 (5.34)	.24	.001
	Post, mean (SD)	4.83 (4.55)	4.95 (5.15)	.72	.000

^a^BCBH-D: Better Choices Better Health Diabetes.

^b^Multiple comparison post-hoc tests following a statistically significant omnibus chi-square test indicate that there are statistically significant differences between the cohorts at the *P*<.05 level (one-tailed).

^c^HbA_1c_: glycated hemoglobin.

However, claims-identified patients did not differ from the rest of the study population regarding depression, HbA_1c_ levels, other comorbid disease burden, years of education, marital status, completion of all study measures, or loss to follow-up. Although there is potential for some biases between the full Lorig et al (2016) study sample and the claims sample based on sex, age, and format of treatment, on the whole, the claims-identified cohort represents the original study population [29].

### Propensity Score Matching Results

The 558 claims-identified patients were 1:3 propensity score matched to a pool of 685,412 patients diagnosed with type II DM and who met the eligibility criteria. The final matched control cohort consisted of 1669 patients. Baseline demographics and health care utilization data for postmatch patients are presented in Table 2. The intervention and matched control cohort had similar demographic characteristics with average age of 55 years, around 65.0% (360/558 and 1097/1669) female, and mostly residing in South (31.0%, 176/558 and 514/1669) and Midwest (42.0%, 231/558 and 697/1669) regions. Both cohorts had similar DCI scores (mean 1.6 [SD 1.2] vs mean 1.6 [SD 1.2]; *P*=.47). As expected, given the propensity score matching process, no significant differences were seen in the prevalence of comorbid illnesses, annual health care utilization, or annual health care costs across the two cohorts at baseline.

### Impact on Comorbid Illness

As can be seen in Table 3, following the intervention, the intervention cohort was associated with significant reductions in medical claims associated with the following comorbid illnesses: hypertension (−50/558, −9.0% vs +10/1669, +0.54%; *P*=.001), hyperlipidemia (−39/558, −7.0% vs −23/1669, −1.44%; *P*=.04), and depression (−16/558, −2.9% vs +12/1669, +0.72%; *P*=.01) compared with the matched control cohort. In addition, the matched control cohort showed significant increases in claims for health care services associated with renal disease (+32/1669, +1.98% vs +1/558, +0.2%; *P*=.006), rheumatoid arthritis or osteoarthritis (+52/1669, +3.12% vs −12/558, −2.2%; *P*=.001), and musculoskeletal disorders (3.72% vs −0.5%, *P*=.04). Thus, there was a decrease in medical claims associated with key comorbid conditions within the intervention cohort from pre- to postintervention period assessment and an increase in claims for conditions among the matched control cohort. An overall composite index that aggregated these 15 diseases for both the pre- and postintervention periods was calculated to capture these shifts in DM-associated comorbid illnesses (eg, an index of 2 means a patient had 2 out of these 15 diseases). During the preintervention period, the matched control’s disease burden (mean 2.0 [SD 1.6]) did not differ statistically from the intervention cohort’s value (mean 2.0 [SD 1.6]; *P*=.86). However, at postintervention period, the matched control’s disease burden (mean 2.1[SD 1.7]) was significantly higher than the intervention cohort’s (mean 1.6 [SD 1.6]; *P*=.001). In addition, the slope of the post- minus preintervention change in disease burden for the intervention cohort was −0.4 (SD 1.5) vs 0.1 (SD 1.4) for the matched control cohort (*P*=.001).

### Health Care Utilization

Change of health care utilization results following the intervention are presented in Table 4. The intervention cohort had reduced utilization of all-cause ED visits and outpatient services compared with the matched control cohort. Although the cohorts showed no significant differences in utilization during the preintervention period, the intervention cohort experienced a significant reduction in all-cause ED visits (−40 per thousand) compared with an increase of 70/1000 for the matched control cohort. Likewise, the intervention cohort had a decrease of 30/1000 inpatient hospitalizations during the follow-up period, whereas the matched control cohort experienced an increase of 10/1000, although this difference did not reach statistical significance (*P*=.10). Finally, the intervention cohort’s use of outpatient services decreased by −5780/1000 contacts during the follow-up compared with a decrease of −290/1000 for the matched control cohort (*P*=.001). For diabetes-related utilization, a statistically significant finding was obtained on outpatient services only.

### Health Care Costs

Health care cost data are presented in Table 5. On the basis of data screening before the main analyses of the cost centers, we noted significant cost outliers in the inpatient and outpatient categories above four SDs of the mean during both the pre- and postintervention assessment periods. For inpatient services, there was one matched control and one intervention patient, each of whom were outliers at preintervention period. At postintervention period, there was one matched control, with inpatient costs above four SDs above the mean. For outpatient costs during the preintervention period, there were two matched control and one intervention cohort members who were above four SDs; at postintervention, there were two matched control and one intervention cohort members who fell into this category. We examined the claims associated with all patients, with inpatient and outpatient costs greater than three SDs above average and found that the most frequently associated primary diagnoses were chronic liver disease and cirrhosis, septicemia, acute kidney failure, acute endocarditis, venous embolism and thrombosis, transient cerebral ischemia, care involving use of rehabilitation procedures, pneumonia, intervertebral disc disorders, disorders of intestine, and venipuncture. On the basis of our review, we capped inpatient costs per year at US $200,000 and outpatient costs at US $150,000. On the whole, this reduced bias against the intervention cohort.

**Table 2:** Baseline demographic and utilization data.

Characteristics		Intervention cohort (N=558)	Matched control cohort (N=1669)	*P* value
Female, n (%)		360 (64.5)	1097 (65.73)	.60
Age (years), mean (SD)		55.29 (8.89)	54.97 (11.19)	.48
**Residence region, n (%)**				
	Northeast	71 9 (12.7)	222 (13.30)	.98
	South	176 (31.5)	514 (30.80)	
	Midwest	231 (41.4)	697 (41.76)	
	West	80 (14.3)	236 (14.14)	
Medicare advantage, n (%)		17 (3.0)	67 (4.01)	.30
**Comorbidities**				
	DCI^a^, (mean, SD)	1.59 (1.19)	1.55 (1.16)	.47
	Hypertension, n (%)	315 (56.5)	945 (56.62)	.94
	Hyperlipidemia, n (%)	315 (56.5)	945 (56.62)	.94
	Obesity, n (%)	62 (11.1)	173 (10.37)	.62
	Chronic obstructive pulmonary disease, n (%)	26 (4.7)	80 (4.79)	.90
	Renal disease, n (%)	13 (2.3)	26 (1.56)	.23
	Depression, n (%)	58 (10.4)	162 (9.71)	.64
	Metabolic syndrome, n (%)	1 (0.2)	4 (0.24)	>.99
	Ischemic heart disease, n (%)	30 (5.4)	82 (4.91)	.66
	Coronary heart disease, n (%)	3 (0.5)	8 (0.48)	>.99
	Peripheral vascular disease, n (%)	11 (2.0)	36 (2.16)	.79
	Osteoporosis, n (%)	7 (1.3)	22 (1.32)	.91
	Rheumatoid arthritis or osteoarthritis, n (%)	50 (9.0)	152 (9.11)	.92
	Low back pain, n (%)	69 (12.4)	223 (13.36)	.55
	Musculoskeletal disorders (low back pain excluded), n (%)	88 (15.8)	269 (16.12)	.85
	Sleep apnea, n (%)	88 (15.8)	248 (14.86)	.60
**Health care utilization, mean (SD)**				
	Hospitalization	0.11 (0.42)	0.1 (0.45)	.60
	ED^b^ visit	0.19 (0.65)	0.19 (0.57)	.83
	Office visit	7.85 (6.41)	7.9 (6.62)	.88
	Lab test	6.14 (4.99)	6.19 (7.03)	.85
**Health care cost (US $), mean (SD)**				
	Total medical cost	7997 (18,045)	8551 (18,235)	.75

^a^DCI: Deyo-Charlson Comorbidity Index.

^b^ED: emergency department.

**Table 3:** Analysis of common comorbid disorders.

Disease	Cohort^a^						Difference in slopes, %	*P* value^b^		
	BCBH-D^c^ intervention^a^ (N=558)			Matched controls^a^ (N=1669)				Difference in slopes	Cohorts time 1	Cohorts time 2
	Pre	Post	Slope, %	Pre	Post	Slope, %				
Hypertension, n (%)	316 (56.6)	266 (47.6)	−9.0	945 (56.62)	955 (57.16)	0.54	−9.5	.001	.98	<.001
Hyperlipidemia, n (%)	316 (56.6)	277 (49.7)	−7.0	945 (56.62)	921 (55.18)	−1.44	−5.5	.04	.98	.03
Obesity, n (%)	62 (11.1)	67 (12.0)	0.9	174 (10.37)	164 (9.77)	−0.60	1.5	.40	.15	.62
Chronic obstructive pulmonary disease, n (%)	26 (4.7)	22 (3.9)	−0.7	80 (4.79)	73 (4.43)	−0.36	−0.4	.71	.90	.62
Renal disease, n (%)	13 (2.3)	14 (2.5)	0.2	27 (1.56)	58 (3.54)	1.98	−1.8	.01	.27	.20
Depression, n (%)	58 (10.4)	42 (7.5)	−2.9	162 (9.71)	174 (10.43)	0.72	−3.6	.01	.63	.03
Metabolic syndrome, n (%)	1 (0.2)	1 (0.2)	0.0	3 (0.24)	8 (0.84)	0.24	−0.2	.64	.78	.22
Ischemic heart disease, n (%)	30 (5.4)	27 (4.8)	0.6	82 (4.91)	103 (6.17)	1.00	−1.5	.25	.67	.22
Coronary heart disease, n (%)	26 (4.7)	22 (3.9)	−0.8	80 (4.79)	78 (4.67)	−0.36	−0.4	.42	.87	.28
Osteoporosis, n (%)	7 (1.3)	6 (1.1)	−0.2	22 (1.32)	42 (2.46)	1.00	−1.2	.17	.91	.02
Rheumatoid arthritis or osteoarthritis, n (%)	50 (9.0)	38 (6.8)	−2.2	152 (9.11)	204 (12.22)	3.12	−5.3	.001	.93	<.001
Lower back pain^d^, n (%)	69 (12.4)	67 (12.0)	−0.4	224 (13.36)	247 (14.80)	1.44	−1.8	.27	.55	.09
Peripheral vascular disease, n (%)	11 (2.0)	12 (2.2)	0.2	37 (2.16)	45 (2.70)	0.54	−0.4	.70	.79	.46
Musculoskeletal disorders^d^, n (%)	88 (15.8)	85 (15.3)	−0.5	269 (16.12)	330 (19.83)	3.72	−4.3	.04	.86	.01
Sleep apnea, n (%)	88 (15.8)	62 (11.1)	−4.7	249 (14.86)	219 (13.12)	1.74	−2.9%	.07	.60	.20
Total disease burden score, mean (SD)	2.0 (1.6)	1.6 (1.6)	−0.4	2.0 (1.6)	2.1 (1.7)	0.1	−0.3	.001	.86	.001

^a^All subjects have 364 days of pretreatment and follow-up time.

^b^Analysis was conducted using generalized estimating equation repeated measures model with a binomial distribution and a logit link. The matched control cohort is the reference category in all analyses.

^c^BCBH-D: Better Choices Better Health Diabetes.

^d^Low back pain is separated out from musculoskeletal disorders.

**Table 4:** Analysis of health care utilization.

Utilization			Cohort^a^														Difference in slopes			*P* value^b^				
			BCBH-D intervention (N=558)							Matched controls (N=1669)										Difference in slopes		Cohorts time 1		Cohorts time 2
			Pre		Post		Slope		Pre			Post		Slope										
**All-cause medical utilization (visits per 1000 members per year)**																			
	Inpatient	110		80		−30		100			110		10		−40			.10			.55		.15	
	Emergency department	190		150		−40		190			260		70		−110			.004			.80		<.001	
	Outpatient	22,880		17,100		–5780		22,880			22,590		−290		–5490			<.001			.99		<.001	
**Diabetes-related medical utilization (visits per 1000 members per year) **																			
	Inpatient	10		0		−10		10			0		−10		0			.57			.75		.71	
	Emergency department	10		0		−10		10			10		0		−10			.27			.91		.30	
	Outpatient	6630		5200		−1430		6760			6530		−230		−1200			<.001			.68		<.001	

^a^All subjects have 1 year of pretreatment time and 1 year of follow-up time.

^b^All analysis were conducted using generalized estimating equation repeated measures model with a logit link and negative binomial distribution for inpatient and emergency department visits and zero-inflated negative binomial distribution for outpatient services. The matched control cohort is the reference category in all analyses.

As Table 5 shows, there were no statistically significant differences in any of the all-cause cost categories during the preintervention period. However, during the postintervention period, the intervention cohort showed significantly lower costs than the matched control cohort for inpatient (*P*=.01), ED services (*P*=.003), and outpatient services (*P*=.01), as well as total all-cause medical costs (*P*=.001). Results for diabetes-specific medical costs presented a different picture from all-cause costs. Diabetes-specific Inpatient and ED cost were low, and no statistically significant findings were obtained between the two cohorts. The cohorts did not have significantly different total all-cause pharmacy costs during the preintervention period (*P*=.34). However, at postintervention period, the matched controls (US $5675) showed higher pharmacy cost than the intervention cohort (US $4264), *P*=.001. There were significant differences in the trajectories of pharmacy costs of the intervention cohort (−US $141) and the matched controls (US $936) over time; *P*=.001.

### Adjusted Cost Savings

Other than the intervention program, the observed costs (in 2016-adjusted US currency) in Table 5 were influenced by patient’s demographic and clinical factors. The maximum likelihood SEM analysis was used to estimate the adjusted cost saving of the program. The starting model for the SEM analysis is shown in Figure 1. This model provided excellent fit to the data (χ^2^_5_=2.5, *P*=.77, CFI=0.997, NFI=0.996, AIC=62.52, RMSE=0.0001 (95% CI 0.00002-0.02). However, none of the regression coefficients from gender to other model variables were statistically significant: preintervention illness burden (*P*=.19), postintervention illness burden (*P*=.69), preintervention total cost (*P*=.14), and postintervention total cost (*P*=.22). Likewise, the regression coefficients from age to preintervention total cost (*P*=.26) and postintervention total cost (*P*=.64) were not significant. Age did show significant effects on preintervention disease burden (0.03 [*P*<.001]) and postintervention disease burden (0.03 [*P*<.001]).

**Figure 2:**
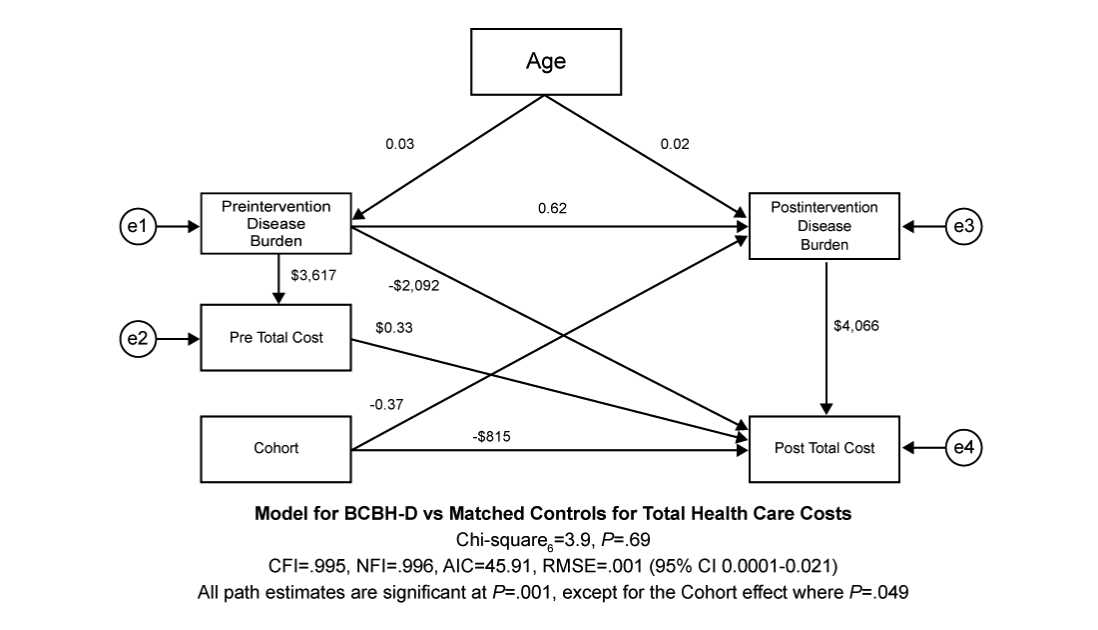
Structural equation model (SEM) final model.

Therefore, gender was removed from the structural model, and age was limited to having effects on pre- and postintervention burden in the trimmed model shown in Figure 2. This final model continued to show excellent fit to the data (χ^2^_6_=3.9, *P*=.69, CFI=0.995, NFI=0.996, AIC=45.91, RMSE=0.001 (95% CI 0.0001-0.02). The final model added one degree of freedom, which allowed us to test if there was a difference in the quality of model fit. A test of the change in the chi-square statistic showed the final model did not show inferior fit compared with the starting model (χ^2^_1_=1.3, *P*>.10), and the comparison of the AIC values indicated the final model to be preferred over the starting model (starting model AIC=62.52 vs final model AIC=45.91).

As Figure 2 illustrates, each additional year of age increased preintervention burden by 0.03 and postintervention burden by 0.02 (*P*=.001 for both). Although age had no direct effects upon postintervention total health care costs, it did have total indirect effects through preintervention burden, postintervention disease burden and preintervention total costs, and postintervention disease burden of US $180. Preintervention disease burden had a direct effect on postintervention disease burden of an increased 0.62 illness (*P*=.001), preintervention total costs of US $3617, and postintervention total costs of US $2092. The indirect effect of preintervention disease burden on postintervention total cost through preintervention total cost was US $1197 and US $2521 through postintervention burden. Thus, the total effects for preintervention disease burden were US $5810. Preintervention total health care costs increased postintervention health care costs by US $0.33 per every preintervention dollar spent during the preintervention period. There were no indirect effects of preintervention total health care costs, so the direct effect equaled the total effect, as illustrated in Figure 2.

**Table 5:** Analysis of health care costs.

Cost (US $)		Cohort^a^							Difference in slopes			*P* value^e^			
		BCBH-D^b^ intervention, medical (N=558)^c^, pharmacy (N=330)^d^			Matched controls, medical (N=1669)^c^, pharmacy (N=990)^d^							Difference in slopes	Cohorts time 1	Cohorts time 2
		Pre	Post	Slope (change in cost)		Pre	Post	Slope (change in cost)						
**All-cause medical cost**														
	Inpatient	2274	1406	−868		2446	2490	44		−912		.03	.78	.01
	Emergency department	381	316	−64		434	502	69		−133		.16	.54	.003
	Outpatient	5327	4040	−1288		5651	5201	−450		−838		.001	.50	.01
	Total	7997	5789	−2207		8551	8213	−338		−1869		.001	.53	.001
**Diabetes-related medical cost**														
	Inpatient	97	124	26		247	74	−172		198		.16	.19	.64
	Emergency department	13	6	−8		18	14	−4		−4		.75	.58	.20
	Outpatient	1437	1249	−189		1943	1711	−232		44		.92	.007	.007
	Total	1558	1383	−175		2223	1808	−415		240		.58	.005	.04
**All-cause pharmacy cost**														
	Total	4405	4264	−141		4739	5675	936		−1078		.001	.34	.001

^a^All subjects have 1 year of pretreatment time and 1 year of follow-up observation time.

^b^BCBH-D: Better Choices Better Health Diabetes.

^c^N of subjects with medical coverage.

^d^N of subjects with pharmacy coverage.

^e^All analyses were conducted using generalized estimating equation repeated measures model with a gamma distribution and a logit link. The matched control cohort is the reference category in all analyses.

The intervention had an indirect association of −0.37 with postintervention disease burden, which had a direct effect of increasing postintervention total health care costs by US $4066. This translated into an indirect effect of –US $1504 for the intervention cohort on postintervention total health care cost through postintervention disease burden. Consequently, the total effects associated with the intervention were –US $2220. After all model adjustments, intervention had a direct effect of reducing postintervention total health care costs by –US $815 (*P*=.049) compared with the matched control cohort as shown in Figure 2.

## Discussion

### Principal Findings

This study shows that beyond the clinical benefits seen in the previous Lorig et al study, participants in the peer-facilitated BCBH-D program experienced reduced all-cause health care utilization and medical costs relative to the matched control group during the year the intervention occurred [29]. The utilization and costs related directly to diabetes care were low to begin with and remained stable over the year of intervention. For diabetes specific care, as documented in the health care utilization and cost results, there was stability, and not worsening, across time. On the other hand, we observed large and significant reductions in all-cause utilization and costs for the intervention cohort for inpatient, ED, and outpatient services, as well as total all-cause medical and pharmacy costs. Intriguingly, there was reduced utilization in the intervention cohort for chronic conditions that often co-occur with DM. Specifically, there were fewer claims for hypertension, hyperlipidemia, and depression among participants in the intervention cohort during the follow-up period, whereas the matched control cohort showed significant increases in claims for health care services associated with renal disease, ischemic heart disease, osteoporosis, and musculoskeletal disorders compared with the intervention cohort. This strengthened the conclusion that a mix of comorbid health issues, many associated with diabetes, were primarily responsible for the all-cause findings. The SEM analysis reinforced this perspective and allowed us to develop significant insight into the systemic effects. Here, we found that postintervention disease burden had a direct effect of increasing postintervention total health care costs by US $4066. The total effects associated with the intervention cohort on postintervention total health care costs were –US $2220 after controlling for all the other variables in the structural model. SEM allowed us to break the total program effects into a –US $815 direct effect component and –US $1504 indirect effect through a postintervention disease burden component. This provides a more accurate estimate of the direct impact of the program’s health care savings.

Even though the study participants were matched on the amount of care they required for comorbid illness in the preintervention period, during the postintervention period, the matched controls required more care for both diabetes-related comorbidities (eg, renal disease and ischemic heart disease) and other conditions (eg, osteoporosis and musculoskeletal disorders). In comparison, the intervention cohort experienced a period in which utilization for hypertension, hyperlipidemia, and depression decreased and care for their other comorbid disorders remained stable. The decreased utilization for depression correlates with one of the main findings of the Lorig et al study that indicated that patients’ self-reported depression was reduced [29]. This may be evidence that the structure or content of the intervention was more successful in decreasing distress and depression associated with diabetes management than the mobile health app studied by Quinn et al [36]. Furthermore, intervention cohort reported increased aerobic activity in the original clinical study, which may correlate with improvements seen in claims for depression and musculoskeletal disorders [29]. It is also plausible that the increased activity may have resulted in weight loss, leading to decreased utilization for hypertension and hyperlipidemia. The successful improvement in physical activity in the intervention cohort adds to the evidence reported by Gibson et al: technology-based strategies can positively impact behavioral measures in patients with diabetes [37].

The generally positive findings reported here need to be put into context with the mixed economic results from DSME reported in other studies. Wertz et al, with a pharmacist-led intervention, found that overall, diabetes-related costs increased to a greater extent in the intervention cohort compared with the control cohort, primarily driven by increased office visits, outpatient visits, and pharmacy claims, whereas costs associated with cardiovascular-related emergency room visits and inpatient visits were significantly higher in the control cohort [38]. This is similar to the findings of Sullivan et al, in which intervention subjects had improved HbA_1c_ values, greater experience of hypoglycemic events, and higher utilization and cost measures after receiving nonspecified diabetes education compared with the control group [21]. The Asheville project, which monitored the 5-year impact of a pharmacist-led diabetes education program, found that total direct medical costs declined while diabetes prescription costs increased; however, there was no control group for comparison [39]. A systematic review of pharmacist-led diabetes education studies by Wang et al showed positive economic cost savings results in the intervention groups compared with usual care [22]. Finally, a study by Burton et al found improvements in clinical measures but no impact on health care utilization or cost [40]. None of these studies are directly analogous to this study. The Wertz study and the Ashville project involved financial incentives that were lacking in this study [38,39]. Burton et al used an eight-session educational program with many possible educational add-ons for a much underserved population [40]. Sullivan et al observed differences between controls and a cohort that had claims codes for any diabetes education but not a specific program [21]. Nevertheless, these studies suggest that not all educational programs are equal, and we have a great deal to learn about intensity of intervention, mode of delivery, use of financial incentives, and population mix before we can make generalized statement about the cost-effectiveness of diabetes self-management education. This is especially evident in the failure of a mobile app, designed for diabetes self-management but lacking a structured educational program, that was noted by Thies et al [41].

The studies that most closely approximate the present observational research are those that utilize computer technology, mobile phone technology or both to disseminate educational and motivational messages. The intervention by Nundy et al was similar to this study in that it had a self-paced education program that lasted for 10 weeks and differed in that it also used a system of reminders and alerts to impact behavior [25]. The control group for Nundy consisted of those who had not responded to invitations to participate in the program, and the possibility of introduced bias was noted by the authors [25]. Similar to our results, positive outcomes were achieved in behavioral, clinical, and economic arenas [25]. The systematic review of computer-based diabetes education programs by Jackson et al, which in general showed positive economic results, had no data regarding hospitalization utilization for internet-based studies but did show a decrease in hospitalization in the computer-based studies, mirroring the success of the intervention by Lorig et al [24,29]. Although several studies are underway that aim to evaluate the impact of technology-mediated diabetes education on cardiovascular comorbidities [42] and cardiovascular risk reduction ([43], results have not yet been published [42,43]. It is important to distinguish that our study is novel in that it reports evidence that comorbidities can be impacted positively.

### Limitations

This study is subject to some specific limitations regarding the sample and more general limitations associated with retrospective analyses of health care claims data. First, we could only identify a portion of the original Lorig et al (2016) study sample because only 82.5% had private insurance [29]. Of those who did have private insurance, we could only find 55% based on name, gender, age, and residence. In addition, some study patients did not meet the 12-month pre- and 12-month postinsurance coverage requirement. Third, it is possible that unobserved patient-specific factors might not be balanced between the Intervention and matched control group, such as health literacy, health coping strategies, life style behaviors, and socioeconomic status, which can impact patients’ access to health promoting resources. As is well known, compliance with interventions, whether physician-directed or self-guided, is always a limitation on effectiveness. Some participants did not attend all the sessions and consequently did not complete the full course of the program. To compensate for this, we used intention-to-treat analysis, which may have conservatively measured the program impact. Finally, our population, from a national sample of commercially insured persons, may not be generalizable to other populations such as a Medicaid population. This potential to create a different result was reflected in Burton et al’s work in an underserved population [40]. Further study in patient groups with different demographic and economic characteristics would be beneficial.

### Conclusions

This study expanded upon the Lorig et al (2016) study by assessing the program’s impact on diabetes-specific and all-cause health care utilization and the cost associated with utilization, as well as shifts in care for common comorbid chronic conditions often observed among patients diagnosed with DM. We found that beyond the clinical benefits seen in the previous Lorig et al report, participants in the peer-facilitated BCBH-D program experienced reduced all-cause health care utilization and medical costs relative to the matched control group [29]. Therefore, we conclude that BCBH-D and other intensive, theory-driven diabetes, self-management programs can produce important clinical changes along with related health care cost savings. The results will require replication in other commercial and noncommercial databases—in particular, the apparent indirect impact of intervention on other disease states will require prospective controlled trial research to verify them.

## Abbreviations

AIC: Akaike Information Criterion
BCBH-D: Better Choices Better Health Diabetes
CFI: Comparative Fit Index
DCI: Deyo-Charlson Comorbidity Index
DM: diabetes mellitus
DSME: Diabetes Self-Management Education
ED: emergency department
GEE: generalized estimating equation
HbA _1c_: glycated hemoglobin
HIRE: HealthCore Integrated Research Environment
ICD-9: International Classification of Diseases-Ninth Revision
IRB: institutional review board
MSA: Metropolitan Statistical Area
NCOA: National Council on Aging
NFI: Normed Fit Index
QALY: quality-adjusted life years
QoL: quality of life
RMSE: root mean square error
SEM: structural equation model

## References

[1] Centers for Disease Control and Prevention (2017). CDC.

[2] American Diabetes Association (2013). Economic costs of diabetes in the US in 2012. Diabetes Care.

[3] Zhuo X, Zhang P, Barker L, Albright A, Thompson TJ, Gregg E (2014). The lifetime cost of diabetes and its implications for diabetes prevention. Diabetes Care.

[4] Piette JD, Kerr EA (2006). The impact of comorbid chronic conditions on diabetes care. Diabetes Care.

[5] Druss BG, Marcus SC, Olfson M, Tanielian T, Elinson L, Pincus HA (2001). Comparing the national economic burden of five chronic conditions. Health Aff (Millwood).

[6] Maddigan SL, Feeny DH, Johnson JA (2005). Health-related quality of life deficits associated with diabetes and comorbidities in a Canadian National Population Health Survey. Qual Life Res.

[7] Wolff JL, Starfield B, Anderson G (2002). Prevalence, expenditures, and complications of multiple chronic conditions in the elderly. Arch Intern Med.

[8] Ciechanowski PS, Katon WJ, Russo JE (2000). Depression and diabetes: impact of depressive symptoms on adherence, function, and costs. Arch Intern Med.

[9] Glasgow RE, Ruggiero L, Eakin EG, Dryfoos J, Chobanian L (1997). Quality of life and associated characteristics in a large national sample of adults with diabetes. Diabetes Care.

[10] Li R, Shrestha SS, Lipman R, Burrows NR, Kolb LE, Rutledge S (2014). Diabetes self-management education and training among privately insured persons with newly diagnosed diabetes--United States, 2011-2012. MMWR Morb Mortal Wkly Rep.

[11] Pantalone KM, Hobbs TM, Wells BJ, Kong SX, Kattan MW, Bouchard J, Yu C, Sakurada B, Milinovich A, Weng W, Bauman JM, Zimmerman RS (2015). Clinical characteristics, complications, comorbidities and treatment patterns among patients with type 2 diabetes mellitus in a large integrated health system. Br Med J Open Diabetes Res Care.

[12] U.S. Department of Health and Human Services Healthypeople.gov.

[13] Pal K, Eastwood SV, Michie S, Farmer A, Barnard ML, Peacock R, Wood B, Edwards P, Murray E (2014). Computer-based interventions to improve self-management in adults with type 2 diabetes: a systematic review and meta-analysis. Diabetes Care.

[14] Sherifali D, Hess R, McTigue KM, Brozic A, Ng K, Gerstein H (2014). Evaluating the feasibility and impact of an internet-based lifestyle management program in a diabetes care setting. Diabetes Technol Ther.

[15] Cotter AP, Durant N, Agne AA, Cherrington AL (2014). Internet interventions to support lifestyle modification for diabetes management: a systematic review of the evidence. J Diabetes Complications.

[16] Palmas W, March D, Darakjy S, Findley SE, Teresi J, Carrasquillo O, Luchsinger JA (2015). Community health worker interventions to improve glycemic control in people with diabetes: a systematic review and meta-analysis. J Gen Intern Med.

[17] Odgers-Jewell K, Ball LE, Kelly JT, Isenring EA, Reidlinger DP, Thomas R (2017). Effectiveness of group-based self-management education for individuals with type 2 diabetes: a systematic review with meta-analyses and meta-regression. Diabet Med.

[18] Lian JX, McGhee SM, Chau J, Wong CK, Lam CL, Wong WC (2017). Systematic review on the cost-effectiveness of self-management education programme for type 2 diabetes mellitus. Diabetes Res Clin Pract.

[19] Mash R, Kroukamp R, Gaziano T, Levitt N (2015). Cost-effectiveness of a diabetes group education program delivered by health promoters with a guiding style in underserved communities in Cape Town, South Africa. Patient Educ Couns.

[20] Odnoletkova I, Ramaekers D, Nobels F, Goderis G, Aertgeerts B, Annemans L (2016). Delivering diabetes education through nurse-led telecoaching. Cost-effectiveness analysis. PLoS One.

[21] Sullivan SD, Dalal MR, Burke JP (2013). The impact of diabetes counseling and education: clinical and cost outcomes from a large population of US managed care patients with type 2 diabetes. Diabetes Educ.

[22] Wang Y, Yeo QQ, Ko Y (2016). Economic evaluations of pharmacist-managed services in people with diabetes mellitus: a systematic review. Diabet Med.

[23] Dalton JE (2008). Web-based care for adults with type 2 diabetes. Can J Diet Pract Res.

[24] Jackson CL, Bolen S, Brancati FL, Batts-Turner ML, Gary TL (2006). A systematic review of interactive computer-assisted technology in diabetes care. Interactive information technology in diabetes care. J Gen Intern Med.

[25] Nundy S, Dick JJ, Chou C, Nocon RS, Chin MH, Peek ME (2014). Mobile phone diabetes project led to improved glycemic control and net savings for Chicago plan participants. Health Aff (Millwood).

[26] Stanford Self-Management Programs (2016). Selfmanagementresource.

[27] Lorig K, Ritter PL, Laurent DD, Plant K, Green M, Jernigan VB, Case S (2010). Online diabetes self-management program: a randomized study. Diabetes Care.

[28] Lorig K, Ritter PL, Turner RM, English K, Laurent DD, Greenberg J (2016). Benefits of diabetes self-management for health plan members: a 6-month translation study. J Med Internet Res.

[29] Lorig K, Ritter PL, Turner RM, English K, Laurent DD, Greenberg J (2016). A diabetes self-management program: 12-month outcome sustainability from a nonreinforced pragmatic trial. J Med Internet Res.

[30] Deyo RA, Cherkin DC, Ciol MA (1992). Adapting a clinical comorbidity index for use with ICD-9-CM administrative databases. J Clin Epidemiol.

[31] Bentler PM, Chou C (1987). Practical issues in structural modeling. Sociol Methods Res.

[32] Hoyle RH (1995). Structural Equation Modeling: Concepts, Issues, and Applications.

[33] Kline RB (2005). Principles and Practice of Structural Equation Modeling. 2nd ed.

[34] SAS Institute (2015). SAS Software, Version 9.3.

[35] StataCorp (2015). Stata Statistical Software, Version Release 14.

[36] Quinn CC, Swasey KK, Crabbe JC, Shardell MD, Terrin ML, Barr EA, Gruber-Baldini AL (2017). The impact of a mobile diabetes health intervention on diabetes distress and depression among adults: secondary analysis of a cluster randomized controlled trial. JMIR Mhealth Uhealth.

[37] Gibson B, Marcus RL, Staggers N, Jones J, Samore M, Weir C (2012). Efficacy of a computerized simulation in promoting walking in individuals with diabetes. J Med Internet Res.

[38] Wertz D, Hou L, DeVries A, Dupclay Jr L, McGowan F, Malinowski B, Cziraky MJ (2012). Clinical and economic outcomes of the Cincinnati Pharmacy Coaching Program for diabetes and hypertension. Manag Care.

[39] Cranor CW, Bunting BA, Christensen DB (2003). The Asheville Project: long-term clinical and economic outcomes of a community pharmacy diabetes care program. J Am Pharm Assoc (Wash).

[40] Burton J, Eggleston B, Brenner J, Truchil A, Zulkiewicz BA, Lewis MA (2017). Community-based health education programs designed to improve clinical measures are unlikely to reduce short-term costs or utilization without additional features targeting these outcomes. Popul Health Manag.

[41] Thies K, Anderson D, Cramer B (2017). Lack of adoption of a mobile app to support patient self-management of diabetes and hypertension in a federally qualified health center: interview analysis of staff and patients in a failed randomized trial. JMIR Hum Factors.

[42] Seto E, Ware P, Logan AG, Cafazzo JA, Chapman KR, Segal P, Ross HJ (2017). Self-management and clinical decision support for patients with complex chronic conditions through the use of smartphone-based telemonitoring: randomized controlled trial protocol. JMIR Res Protoc.

[43] Lynch CP, Williams JS, Ruggeiro KJ, Knapp RG, Egede LE (2016). Tablet-aided behavioral intervention effect on self-management skills (TABLETS) for diabetes. Trials.

